# Intraoperative pleth variability index-based fluid management therapy and gastrointestinal surgical outcomes in elderly patients: a randomised controlled trial

**DOI:** 10.1186/s13741-023-00308-0

**Published:** 2023-05-12

**Authors:** Yu Wang, Yue Zhang, Jin Zheng, Xue Dong, Caineng Wu, Zhijia Guo, Xinhai Wu

**Affiliations:** 1 Department of Anaesthesiology, Peking University Shenzhen Hospital, Shenzhen, 518036 China; 2 Clinical Research Institute, Shenzhen-Peking University, The Hong Kong University of Science & Technology Medical Center, Shenzhen, China; 3 Department of Anaesthesiology, Yuebei People’s Hospital, Shaoguan, China; 4 Department of Anesthesiology, The First Affiliated Hospital of Guangzhou University of Chinese Medicine, Guangzhou, China; 5 Department of Anaesthesiology, First Hospital of Shanxi Medical University, Taiyuan, China

**Keywords:** Gastrointestinal surgery, Goal-directed fluid therapy, Pleth variability index, Elderly patients, Postoperative complications, Dual-centre trial, Randomised controlled trial

## Abstract

**Background:**

Intraoperative goal-directed fluid therapy (GDFT) has been reported to reduce postoperative complications of patients undergoing major abdominal surgery. The clinical benefits of pleth variability index (PVI)-directed fluid management for gastrointestinal (GI) surgical patients remain unclear. Therefore, this study aimed to evaluate the impact of PVI-directed GDFT on GI surgical outcomes in elderly patients.

**Methods:**

This randomised controlled trial was conducted in two university teaching hospitals from November 2017 to December 2020. In total, 220 older adults undergoing GI surgery were randomised to the GDFT or conventional fluid therapy (CFT) group (*n* = 110 each). The primary outcome was a composite of complications within 30 postoperative days. The secondary outcomes were cardiopulmonary complications, time to first flatus, postoperative nausea and vomiting, and postoperative length of stay.

**Results:**

The total volumes of fluid administered were less in the GDFT group than in the CFT group (2.075 L versus [vs.] 2.5 L, *P* = 0.008). In intention-to-treat analysis, there was no difference in overall complications between the CFT group (41.3%) and GDFT group (43.0%) (odds ratio [OR] = 0.935; 95% confidence interval [CI], 0.541–1.615; *P* = 0.809). The proportion of cardiopulmonary complications was higher in the CFT group than in the GDFT group (19.2% vs. 8.4%; OR = 2.593, 95% CI, 1.120–5.999; *P* = 0.022). No other differences were identified between the two groups.

**Conclusions:**

Among elderly patients undergoing GI surgery, intraoperative GDFT based on the simple and non-invasive PVI did not reduce the occurrence of composite postoperative complications but was associated with a lower cardiopulmonary complication rate than usual fluid management.

**Trial registration:**

This trial was registered with the Chinese Clinical Trial Registry (ChiCTR-TRC-17012220) on 1 August 2017.

## Background

The number of elderly patients undergoing surgical procedures is growing drastically with the ageing of society. This population is at a greater risk of mortality and morbidity after gastrointestinal (GI) surgical procedures than younger patients. Despite advances in surgical and perioperative care, postoperative complications occur in approximately 45% of patients, with an in-hospital mortality rate of approximately 3% in elderly patients undergoing GI surgical procedures (Pearse et al. 2014). To minimise these risks, anaesthesiologists try to optimise intraoperative haemodynamics and fluid management in these patients.

Fluid deficits are frequent during GI procedures because of preoperative fasting, bowel preparation, and intraoperative fluid loss, particularly in elderly patients (Holte et al. 2002). Therefore, intraoperative fluid management is critical for anaesthetic practice in these procedures (Lobo et al. 2002; Junghans et al. 2006). Individualised goal-directed fluid therapy (GDFT) has been reported to improve oxygen delivery and overall haemodynamic function, thereby reducing postoperative complications in high-risk patients (Gan et al. 2002; Corcoran et al. 2012; Hamilton et al. 2011). GDFT has been performed with different haemodynamic monitoring devices to guide intravenous fluid administration in high-risk surgical patients (Sandham et al. 2003; Challand et al. 2012; Lopes et al. 2007; Benes et al. 2010; Malbouisson et al. 2017; Bloria et al.^2022^). However, data on the optimal haemodynamic parameters and devices for elderly patients undergoing GI surgery are limited. Most of these monitoring devices are invasive and not routinely available in daily clinical practice. A non-invasive ‘plug and play’ sensor (Masimo Corporation, Irvine, CA, USA) that uses the pleth variability index (PVI) was developed to assess fluid responsiveness based on plethysmographic variations induced by mechanical ventilation. The PVI has been shown to perform similarly to more invasive and expensive dynamic fluid assessment technologies (such as pulse pressure variation and stroke volume variation) during cardiac surgery (Cannesson et al. 2008; Haas et al. 2012), colorectal surgery (Hood and Wilson 2011), bariatric surgery (Demirel et al. 2018). A small-sized randomised controlled trial (RCT) showed that PVI-directed fluid management reduced the lactate concentrations and improved fluid management in abdominal surgery recipients (Forget et al. 2010). However, its clinical benefits for elderly GI surgical patients remain unclear.

This dual-centre RCT primarily aimed to analyse whether perioperative GDFT based on the PVI resulted in decreased composite postoperative complications compared with conventional fluid management practice in elderly patients who underwent major GI surgery. The secondary objective of this RCT was to evaluate cardiopulmonary major postoperative complications, postoperative nausea and vomiting (PONV), time to first flatus, and postoperative length of stay (PLOS).

## Methods

### Study design and ethics statements

This dual-centre clinical RCT was conducted in two university teaching hospitals from November 2017 to December 2020 (Peking University Shenzhen Hospital and First Hospital of Shanxi Medical University) The study analysed whether perioperative GDFT based on PVI reduced composite postoperative complications in elderly patients who underwent major GI surgery compared to conventional fluid management practice It also evaluated cardiopulmonary major postoperative complications, PONV, time to first flatus, and PLOS. This study was approved by the Clinical Research Ethics Committee of Peking University Shenzhen Hospital (IRB 2017-001-2), and written informed consent was obtained from all participants in the trial. Prior to patient enrolment, this trial was registered with the Chinese Clinical Trial Registry (ChiCTR-TRC-17012220, principal investigator: Xinhai Wu, date of registration: 1 August 2017). This manuscript adheres to the applicable guidelines of the Consolidated Standards of Reporting Trials (CONSORT).

#### Inclusion criteria

Potential participants were screened on the day before surgery (or on the preceding Friday for those who underwent surgery on a Monday). Adult patients aged ≥ 65 who underwent elective major GI surgery (including gastrectomy, small bowel resection, and colorectal surgery) were considered eligible.

#### Exclusion criteria

Patients were excluded if they met any of the following criteria: (1) history of severe cardiac disease, including severe arrhythmia, myocardial infarction, and cardiac insufficiency; (2) presence of hepatorenal dysfunction; (3) presence of cardiac failure (New York Heart Association class III or IV); (4) history of asthma; (5) presence of pulmonary infection; and (6) body mass index ≥ 30 kg/m^2^.

#### Data collection

Detailed information, including baseline demographic data, preoperative medical history, diagnosis at the time of admission, illness severity and perioperative variables were obtained after recruitment. After obtaining written informed consent, baseline data (including demographic data, surgical diagnosis, and comorbidities) were collected.

### Randomisation and blinding

A biostatistician not involved in the data management and statistical analyses generated random numbers (at a 1:1 ratio, stratified by centre) using SAS software version 9.2 (SAS Institute, Cary, NC, USA), with a block size of 4. Results of this randomisation were sealed in sequentially numbered envelopes and kept until the end of the study by a study coordinator (J.Z.) who was not involved in data collection, perioperative care, or postoperative follow-up. During the study period, consecutively recruited patients received either intraoperative GDFT or conventional haemodynamic management, according to the random number allocation by the study coordinator (J.Z.). Intraoperative data of each recruited patient were collected by anaesthesiologists (Y.W, X.D, Z.G, and J.Z). Anaesthesiologists and investigators did not communicate with each other regarding the patient data collected. Both patients and postoperative investigators were blinded to the study group assignment. Unblinding was conducted after the trial was closed and data of all patients were collected. Older adults undergoing GI surgery were randomly assigned to the GDFT or conventional fluid therapy (CFT) group.

### Technical information

#### Interventions, anaesthesia, and perioperative care

No premedication was administered, and solid food and clear fluid intakewere allowed until 8 h and 2 h before surgery, respectively. Patients’ electrocardiographic activity, arterial blood pressure, oxygen saturation, and body temperature were continuously monitored. General anaesthesia was induced with etomidate or propofol and sufentanil; atracurium or rocuronium was used for neuromuscular block. A 20-gauge radial artery catheter and central venous access catheter were inserted at the end of the induction phase. Anaesthesia was maintained with sevoflurane or propofol and remifentanil. The PVI sensor (Rainbow R2-25a, Masimo Corporation, Irvine, CA, USA) was placed on the patient’s index finger to monitor the PVI continuously.. The use of additional regional anaesthesia, choice of anaesthetic drugs, and operative pain management were at the discretion of the attending anaesthesiologist. The investigators indicated that the ventilator patterns should be restricted as follows: controlled ventilation with a tidal volume of 8 mL/kg of ideal body weight; an initial respiratory rate of 12 breaths/min adjusted to achieve end-tidal CO_2_ between 35 and 45 mmHg; and pulse oxygen saturation > 96%. In all cases, the anaesthetic procedure was chosen by the attending anaesthesiologist. Packed red blood cells were administered at the anaesthesiologist’s discretion (our perioperative care protocol only suggested using haemoglobin levels of 7 g/dL as a threshold for healthy patients and 9 g/dL for patients with pulmonary or cardiac diseases). Lactate concentrations at the beginning of surgery and at discharge from the post-anaesthesia care unit (PACU) were analysed.

#### Intraoperative haemodynamic protocol

**Fig. 1:**
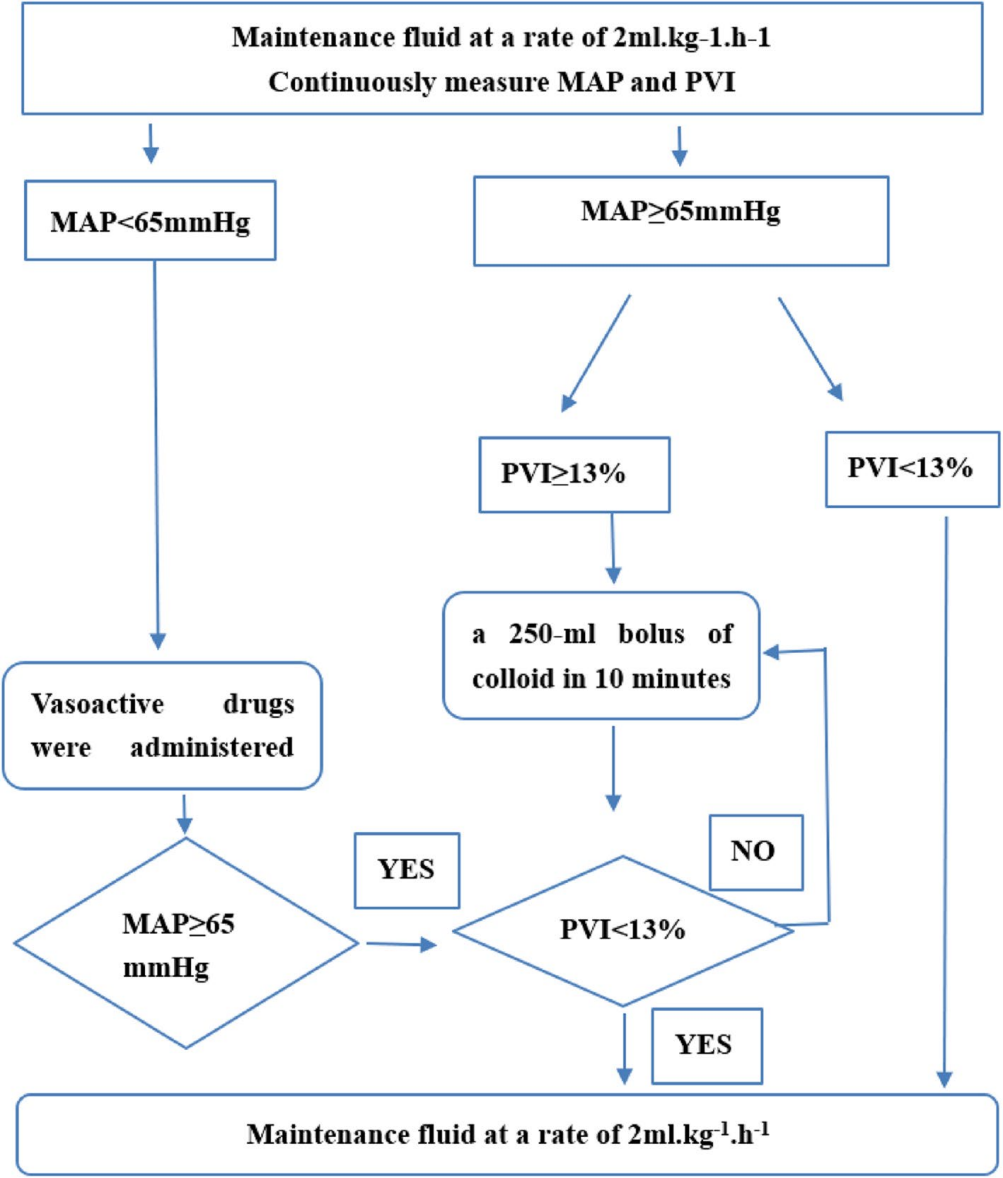
Intraoperative fluid treatment in the intervention groups. MAP, mean arterial pressure; PVI, pleth variability index

In the GDFT group, 500 mL of crystalloids were infused during induction, followed by continuous infusion at a rate of 2 mL·kg^−1.^h^−1^. When MAP is < 65 mmHg, the vasoactive drugs((phenylephrine, ephedrine, or norepinephrine)) were promptly administered to maintain a mean arterial blood pressure of ≥ 65 mmHg regardless of PVI. When MAP is ≥ 65 mmHg, we judge fluid responsiveness is present via PVI > 13%. Whenever the PVI was > 13% for 5 min, we administered a 250-mL bolus of colloid (6% hydroxyethyl starch, Voluven®, Fresenius Kabi, Beijing, China). The dose was repeated every 5 min until the PVI was < 13% (Fig. 1).

In the CFT group, 500 mL of crystalloid was infused during induction, followed by a continuous infusion of crystalloids (6 mL·kg^−1.^h^−1^). A bolus of 250-mL colloids(6% hydroxyethyl starch, Voluven®, Fresenius Kabi, Beijing, China) was administered whenever acute blood loss was > 50 mL, the mean arterial blood pressure decreased to < 65 mmHg, or the central venous pressure decreased to < 5 mmHg. A repeat bolus was administered after waiting for 5 min if any one of these criteria was met. Whenever the mean arterial blood pressure decreased to < 65 mmHg and remained unresponsive to fluids, vasoactive drugs (phenylephrine, ephedrine, or norepinephrine) were administered to maintain a mean arterial blood pressure of > 65 mmHg.

### Outcome assessment

The primary outcome assessed was the proportion of patients who experienced clinically detected postoperative complications at 30 postoperative days, including surgical site infection, organ/space infection, anastomotic leakage, new-onset stroke, confusion/delirium, pneumonia, atelectasis, pleural effusion requiring drainage, arrhythmias, acute myocardial infarction, acute kidney injury, reoperation for bleeding, pulmonary embolism, deep vein thrombosis, paralytic ileus, and mortality within 30 days after surgery. The postoperative complications were based on standard definitions for cardiovascular, respiratory, abdominal, renal, and central nervous complications as well as relevant infections similar to previous studies (Pearse et al. 2014; Szturz et al. 2019).

We assessed the following secondary outcomes: cardiopulmonary complications included pneumonia, atelectasis, pulm oedema, arrhythmia and acute myocardial infarction, PONV, time to first flatus, and PLOS.

Data were recorded in a case report file maintained by the principal investigator at each centre and stored in the REDCap database (Vanderbilt University, Nashville, TN, USA). Data were obtained from the clinical files completed by the surgeons responsible for the patient, but who were blinded to the study. Data validation (conformity between the case report file and database, screening for internal coherence of recorded values, and detection of abnormalities and discrepancies according to the plan of controls previously prepared) were performed by the principal investigator (X-H.W.).

### Statistical analysis

Based on previous published data (Pearse et al. 2014) and past patient data available in our hospital, we estimated that complications might appear in 45% of patients; therefore, we considered a reduction from 45 to 35% as clinically relevant. Assuming a two-sided type I error rate of 5% and a power of 70%, the ideal sample size was determined to be 196 patients. Considering a sample loss rate of approximately 10%, we were required to enrol 110 patients for each group. The sample size was calculated using Stata 13.1 (StataCorp, College Station, TX, USA).

Unless otherwise indicated, all results are expressed as mean and standard deviation or as the median and interquartile range (IQR). Normally distributed continuous data were compared using the independent-samples *t*-test, whereas non-normally distributed continuous data were compared using the independent-samples Mann–Whitney U test. Categorical variables were analysed using the χ^2^ test, continuity correction χ^2^ test, or Fisher exact test; two-tailed tests were performed whenever appropriate, and a *P*-value of < 0.05 was considered statistically significant. All statistical analyses were performed using SPSS Statistics for Windows, version 27.0 (IBM Corp., Armonk, NY, USA).

## Results

### Participant flow and recruitment

**Fig. 2:**
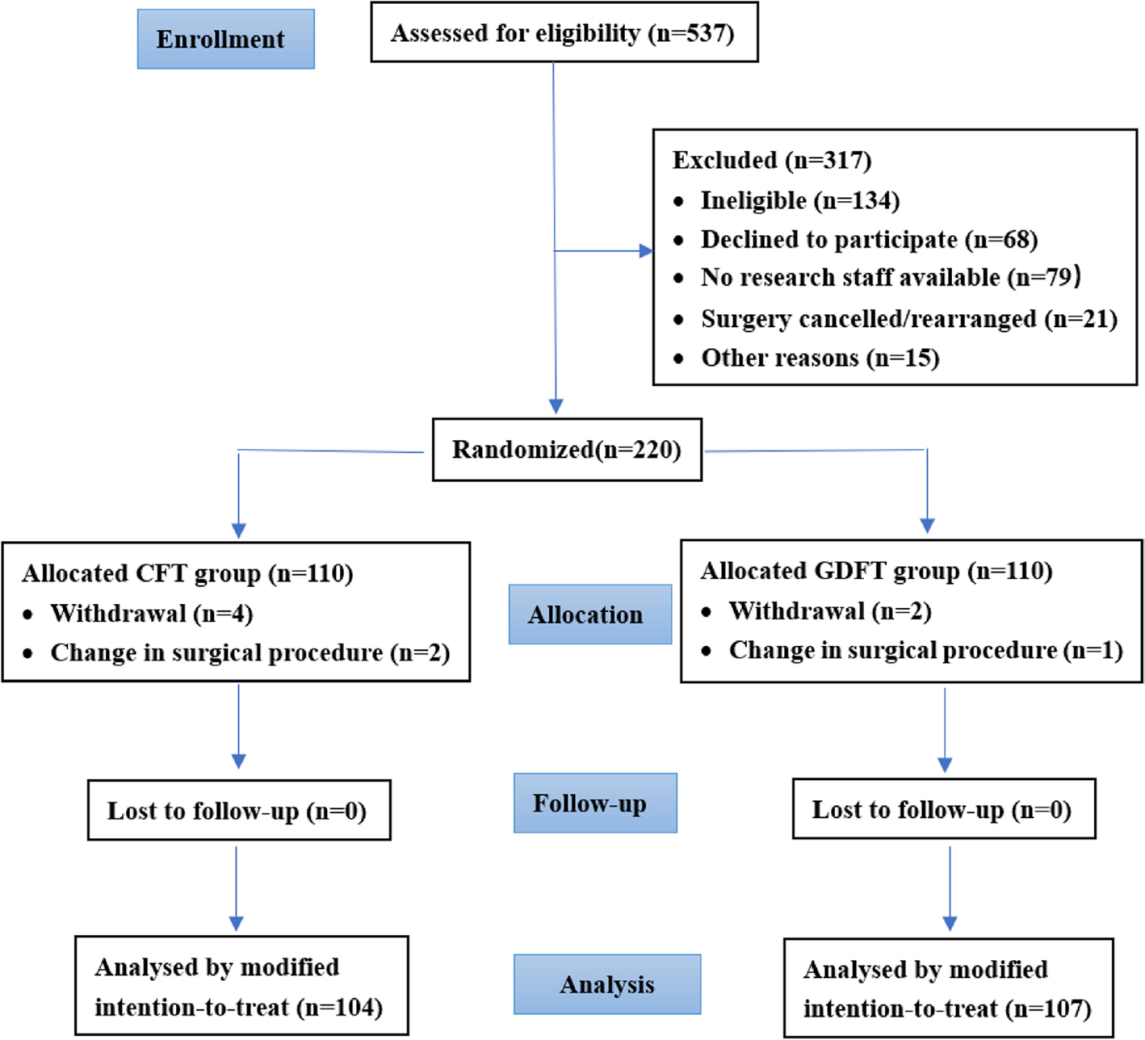
Consolidated Standards of Reporting Trials (CONSORT) diagram. CFT, conventional fluid therapy; GDFT, goal-directed fluid therapy; ITT, intention to treat

From 5 November 2017 to 29 October 2020, 537 patients were eligible for enrolment, and 220 patients from two hospitals (160 participants from the Peking University Shenzhen Hospital and 60 participants from the First Hospital of Shanxi Medical University) were enrolled and randomised. The final follow-up of the last randomised participants was performed in December 2020. Of 220 patients, 9 were excluded after randomisation based on the protocol-defined exclusion criteria: 6 patients withdrew from the study, and 3 patients were excluded because of a change in the surgical procedure. All evaluable patients were followed up for 30 days postoperatively, and none were lost to follow-up. A total of 211 patients were included in the intention-to-treat analyses (CFT group, 104; GDFT group, 107) (Fig. 2). Results were reported according to the CONSORT guidelines.

### Baseline data

There were no between-group differences in patient characteristics, physical status according to the American Society of Anaesthesiologists guidelines, comorbidities, preoperative haemoglobin level, and creatinine level (Table 1). The overall median age was 70 years (IQR, 68–79 years).

**Table 1:** Baseline characteristics

	CFT (*n* = 104)	GDFT (*n* = 107)	*P*-value
Age (years)	70.7 ± 5.2	70.7 ± 5.4	0.859
Male sex (%)	72 (69.2)	73 (68.2)	0.875
BMI, kg/m^2^	22.9 ± 3.2	22.4 ± 3.0	0.253
ASA physical status,* n* (%)			
I	7 (6.7)	12 (11.2)	
II	79 (56.4)	80 (74.8)	
III	18 (17.3)	15 (8.8)	0.460
Smoker, *n* (%)	12 (11.5)	12 (11.2)	0.941
Chronic alcohol consumption, *n* (%)	5 (4.8)	4 (3.7)	0.701
Medical history, *n* (%)			
Pulmonary disease	1 (0.9)	3 (2.8)	0.634
Cardiovascular disease	39 (37.5)	36 (33.6)	0.929
Diabetes mellitus	14 (13.5)	22 (20.6)	0.171
Cerebral vascular disease	3 (2.9)	5 (4.7)	0.749
Preoperative haemoglobin level, g/dL	13.5 ± 1.3	13.4 ± 1.6	0.878
Preoperative creatinine level, mmol/L	71.3 ± 13.4	82.0 ± 21.9	0.675

Continuous data are expressed as mean ± standard deviation or median (interquartile range). Categorical data are expressed as the number of patients (percentage). Statistical significance was tested by the t-test, Mann–Whitney test, and Fisher exact test

*CFT* Conventional fluid therapy, *GDFT* Goal-directed fluid therapy, *BMI* Body mass index, *ASA* American Society of Anaesthesiologists

### Intraoperative data

The surgery types and surgical methods were similar between the CFT and GDFT groups (*P* = 0.280 and *P* = 0.254, respectively). The duration of surgery and anaesthesia was not significantly different between the two groups (235 [190, 290] min versus [vs.] 202 [175, 270] min and 290 [230, 345] min vs. 257 [210, 313] min; *P* = 0.093 and* P* = 0.096, respectively). The total volumes of fluid administered and crystalloid infusion were lesser in the GDFT group than in the CFT group (2075 [1900, 2600] mL vs. 2500 [2000, 3100] mL, and 1450 [1200, 2100] mL vs. 1795 [1500, 2187] mL; *P* = 0.008 and *P* = 0.009, respectively). Lactate concentrations at the beginning of the surgery (0.9 [0.7, 1.1] mEq/L vs. 1.0 [0.7, 1.4] mEq/L, *P* = 0.675) and at discharge (0.9 [0.7, 1.1] mEq/L vs. 1.1 [0.8, 1.5] mEq/L; *P* = 0.733) from the PACU were similar between the CFT and GDFT groups (Table 2).

**Table 2:** Intraoperative data

	CFT (*n* = 104)	GDFT (*n* = 107)	*P-*value
Type of surgery,* n* (%)			
Colon resection	49 (47.1)	40 (37.4)	
Rectum resection	26 (25.0)	36 (33.6)	
Gastric resection	29 (27.9)	31 (29.0)	0.280
Surgical method, *n* (%)			
Open	23 (22.1)	31 (29.0)	
Laparoscopic	81 (77.9)	76 (71.0)	0.254
Duration of surgery (min)	235 (190, 290)	202 (175, 270)	0.093
Anaesthesia time (min)	290 (230, 345)	257 (210, 313)	0.096
Total volume of fluid administered (mL)	2500 (2000, 3100)	2075 (1900, 2600)	0.008*
Crystalloid infusion (mL)	1795 (1500, 2187)	1450 (1200, 2100)	0.009*
Colloid infusion (mL)	500 (500, 1000)	500 (500, 500)	0.308
Estimated blood loss (mL)	100 (30, 200)	50 (30, 200)	0.410
Patients receiving a blood transfusion, *n* (%)	19 (18.3)	13 (12.1)	0.214
Urine output (mL)	400 (300, 600)	400 (200, 500)	0.350
Patients receiving a vasoactive infusion, *n* (%)	50 (48.1)	44 (41.1)	0.295
Lactate before skin incision, mEq/L	0.9 (0.7, 1.1)	1.0 (0.7, 1.4)	0.675
Lactate at PACU departure, mEq/L	0.9 (0.7,1.1)	1.1 (0.8, 1.5)	0.733

Continuous data are expressed as mean ± standard deviation or median (interquartile range). Categorical data are expressed as the number of patients (percentage). Statistical significance was tested by the t-test, Mann–Whitney test, and Fisher exact test

*CFT* Conventional fluid therapy, *GDFT* Goal-directed fluid therapy, *PACU* Post-anaesthesia care unit

^*^Statistically significant difference

### Outcomes

The number of patients with one or more complications at 30 days was similar between the CFT group (43 [41.3%]) and GDFT group (46 [43.0%]) (odds ratio [OR] = 0.935; 95% confidence interval [CI], 0.541–1.615; *P* = 0.809). The proportion of cardiopulmonary complication was higher in the CFT group than in the GDFT group (19.2% vs. 8.4%; OR = 2.593, 95% CI, 1.120–5.999; *P* = 0.022). No significant between-group differences in PONV, the time to first flatus, and PLOS were identified (*P* = 0.398, *P* = 0.475, and *P* = 0.614, respectively) (Table 3).

**Table 3:** Postoperative outcomes

	CFT (*n* = 104)	GDFT (*n* = 107)	*P*-value	OR (95% CI)
**Primary outcome**				
Number of patients with one or more complications, *n* (%)	43 (41.3)	46 (43.0)	0.809	0.935 (0.541–1.615)
Individual elements				
Surgical site infection, *n* (%)	6 (5.8)	3 (2.8)	0.468	
Organ/space infection,* n* (%)	3 (2.9)	2 (1.9)	0.974	
Other infection, *n* (%)	3 (2.9)	3 (2.8)	> 0.999	
Anastomotic leakage, *n* (%)	5 (4.8)	1 (0.9)	0.201	
New-onset stroke, *n* (%)	0 (0)	1 (0.9)	> 0.999	
Confusion/delirium, *n* (%)	3 (2.9)	3 (2.8)	> 0.999	
Pneumonia, *n* (%)	6 (5.8)	2 (1.9)	0.262	
Atelectasis, *n* (%)	2 (1.9)	0 (0)	0.242	
Pulmonary oedema, *n* (%)	5 (4.8)	2 (1.9)	0.420	
Arrhythmia, *n* (%)	5 (4.8)	2 (1.9)	0.420	
Acute myocardial infarction, *n* (%)	2(1.9)	3 (2.8)	> 0.999	
Reoperation for bleeding, *n* (%)	1 (0.9)	1 (0.9)	> 0.999	
Acute kidney injury, *n* (%)	3 (2.9)	5 (4.7)	0.749	
Pulmonary embolism, *n* (%)	0 (0)	0 (0)	> 0.999	
Deep vein thrombosis, *n* (%)	2 (1.9)	1 (0.9)	0.980	
Paralytic ileus, *n* (%)	15 (14.4)	20 (18.7)	0.405	
Mortality, *n* (%)	2 (1.9)	1 (0.9)	0.980	
**Secondary outcome**				
Cardiopulmonary complication, n (%)	20 (19.2)	9 (8.4)	0.022*	2.593 (1.120–5.999)
PONV, n (%)	35 (33.7)	42 (39.3)	0.398	0.785 (0.447–1.377)
Time to first flatus (h)	52 (34, 81)	60 (30, 93)	0.475	
PLOS (days)	10 (8, 12)	9(8, 14)	0.614	

Continuous data are expressed as mean ± standard deviation or median (interquartile range). Categorical data are expressed as the number of patients (percentage). Statistical significance was tested by the t-test, Mann–Whitney test, and Fisher exact test

*CFT* Conventional fluid therapy, *GDFT* Goal-directed fluid therapy, *PONV* Postoperative nausea and vomiting, *PLOS* Postoperative length of stay, *OR* Odds ratio, *CI* Confidence interval

^*^Statistically significant difference

## Discussion

This study examined the impact of intraoperative GDFT based on the non-invasive PVI on GI surgical outcomes in elderly patients. The principal finding of our trial was that among elderly patients undergoing major GI surgery, PVI-directed intraoperative GDFT was not associated with a significant reduction in the proportion of patients who died or experienced complications within 30 postoperative days, compared with CFT. The analysis of secondary outcomes revealed that the intervention reduced cardiopulmonary complications but did not provide clinical benefits in terms of PONV, time to first flatus, and PLOS compared with usual fluid management.

One of the reported effects of PVI-directed GDFT was the decreased volume of fluid infusion required intraoperatively, with no effect on lactate levels (Demirel et al. 2018; Fischer et al. 2020). However, the difference in fluid administration between the GDFT and standard care groups was varied in numerous GDFT trials. A meta-analysis of 56 GDFT studies reported that the differences were within 500 mL in 35 (62%) trials, > 500 mL in 10 (18%) trials, and < 500 mL in 11 (20%) trials (Jessen et al. 2022). Greater volume difference was shown in earlier studies because of adoption of liberal fluid therapy in their control group (Sandham et al. 2003; Malbouisson et al. 2017; Lopes et al. 2007; Benes et al. 2010). With enhanced recovery after surgery (ERAS) programmes and laparoscopic procedures being widely implemented, restricted fluid therapy has been mostly used for intraoperative conventional fluid administration, recently. In our study, the difference of total fluid administration between the CFT and GDFT groups was 425 mL, which was consistent with most findings in the literature. Nonetheless, the difference in fluid administration in the CFT and GDFT groups was not as significant as expected. This could partly explain why, in the primary analysis, GDFT based on the PVI was not found to reduce the occurrence of composite postoperative complications.

Previous studies have shown that GDFT based on different invasive haemodynamic parameters can reduce postoperative complication rates and shorten PLOS in high-risk surgery (Sandham et al. 2003; Challand et al. 2012; Lopes et al. 2007; Benes et al. 2010; Szturz et al. 2019). However, the overall certainty in the evidence was low because of the heterogeneity of the studies (Jessen et al. 2022). Our findings are not as conclusive as the results of these previous studies. We believe that the differences in the research findings can be attributed to the heterogeneous populations, varying surgeries, and inconsistencies in the implementation of fluid management protocols. In addition, with the improvement of surgical techniques and the popularisation of ERAS, the incidence of postoperative complications has decreased remarkably. Therefore, the potential effect of GDFT to reduce postoperative complications is inapparent under the current ERAS clinical path (Rollins and Lobo 2016; Gómez-Izquierdo et al. 2017).

Interestingly, in our secondary outcomes assessment, reduction was noted in the incidence of cardiopulmonary complications in the GDFT group compared with the CFT group (8.4% vs. 19.2%). The mechanism of the beneficial effect of GDFT could not be determined in our study. It is possible that elderly patients receiving GDFT benefit from fluid optimisation and avoid excessive fluid intake and, therefore, have a decreased risk of overload of the heart and tissue oedema, which, compared to CFT, could have potentially lowered the risk of postoperative complications such as pulmonary oedema (1.9% vs. 4.8%), arrhythmia (1.9 vs. 4.8%), pneumonia (1.9% vs. 5.8%), surgical site infection (2.8% vs. 5.8%), and anastomotic leakage (0.9% vs. 4.8%). A recent meta-analysis of GDFT trials concluded that GDFT during general anaesthesia might reduce pneumonia, surgical site infection, and anastomotic leakage, and this result reached moderate certainty in the evidence (Jessen et al. 2022). However, RCTs with greater sample sizes are warranted to accurately demonstrate statistical differences because of the low incidence of these postoperative complications.

Recovery of GI function is a significant determinant of in-hospital recovery after GI surgery (Augestad and Delaney 2010). Postoperative GI disturbance is a common complication manifested by delayed intestinal motility and PONV. Individualised GDFT guided by haemodynamic parameters seems to be the logical approach to avoid inappropriate intestinal perfusion, which can lead to postoperative GI dysfunction. In our trial, the occurrence rate of paralytic ileus and time to first flatus were similar between the two groups. GDFT was not found to improve postoperative GI function of elderly patients undergoing GI surgery. There is conflicting evidence on whether optimising fluids management can reduce the risk of PONV. In an earlier study, Gan et al. (2002) reported that GDFT guided by oesophageal Doppler monitoring results in an earlier return to bowel function and a lower incidence of PONV. In a meta-analysis, Jewer et al. (2019) concluded that supplemental intravenous crystalloid administration prevents PONV in patients undergoing surgical procedures under general anaesthesia. In our trial, there were more patients with PONV in the GDFT group (39.3%) than in the CFT group (33.7%), although this difference was not statistically significant. Large sample studies are needed to assess the risk-benefit profile of fluid therapy and PONV.

This study has some limitations. First, GDFT was performed only intraoperatively. Therefore, it is unclear if the intraoperative assessment of fluid administration is adequate to produce a considerable difference in the outcome compared to the perioperative assessment. The use of GDFT throughout the perioperative period may be more effective in improving patient outcomes. Second, the cut-off value for the PVI was a limitation. The threshold value of the PVI in our interventional group was set at 13% based on that reported in previous literature (Cannesson et al. 2008). A recent meta-analysis showed high variability regarding the best threshold value, ranging from 7 to 20% (Perel 2014). One reason for the high variability may be differences in clinical settings and parameters of the study population, including age, vasoactive drug use, position during surgery, and pneumoperitoneum. Therefore, more studies are warranted to verify the optimum threshold value according to the study settings and participant population. Third, given the nature of GDFT, it is practically impossible to blind the clinical team performing the intervention. This may lead to potential research bias. Fourth, our sample size is small, and the power of the trial is low (70%) due to time and financial constraints. Increasing the power may led to different results.

## Conclusions

Among elderly patients undergoing GI surgery, intraoperative GDFT based on the PVI showed no superiority to the conventional fluid management regimen in terms of overall complications, PONV, and PLOS, but was associated with a lower cardiopulmonary complication rate than usual fluid management.

## Data Availability

The datasets generated and/or analysed during the current study are available in the REDcap database (http://202.105.127.142:81/).

## Abbreviations

CFT: Conventional fluid therapy
CI: Confidence interval
GDFT: Goal-directed fluid therapy
GI: Gastrointestinal
OR: Odds ratio
PACU: Post-anaesthesia care unit
PLOS: Postoperative length of stay
PONV: Postoperative nausea and vomiting
PVI: Pleth variability index
vs.: Versus
